# A new approach for aggregation of *Paramecium caudatum* by nitric oxide

**Published:** 2013-03

**Authors:** Manizheh Karami, Seyed Sajad Shahrokhi, Bahram Kazemi, Seyedeh Samaneh Moezzi

**Affiliations:** 1 Department of Biology, Faculty of Basic Sciences, Shahed University, Tehran, Iran; 2 Biotechnology & Molecular Biology Research Center, Shahid Beheshti University of Medical Sciences, Tehran, Iran

**Keywords:** Morphine, Nitric oxide, NADPH-diaphorase, Aggregation, *Paramecium*

## Abstract

**Background and Objectives:**

Nitric oxide (NO) plays a role in thermoregulation and growth of protozoa. This work aimed to add the molecule NO in physiology of protozoa in contact with abused narcotic substances.

**Materials and Methods:**

A sedative drug, morphine, was infused into a cell chamber containing *Paramecia*. The cell response to the drug was recorded promptly after drug infusion using a potency protocol provided for the first time at this laboratory. A precursor of NO, L-arginine, was treated jointly with drug to involve the NO system in protozoan performance to drug exposure. Marking of NADPH-diaphorase (NADPH-d) was followed to provide data to explain the mechanisms.

**Results:**

Morphine particularly (0.5 to 60 µg/µ1) aggregated the *Paramecia*. The infusion of L-arginine (1 to 8 µg/µ1) together with morphine potentiated this effect, though, pre-usage of L-NAME (1 to 8 µg/µ1), a blocker of NO production, reversed the response. Notably the activation of NADPH-d in solely morphine or L-arginine plus morphine samples was revealed. However, the expression of marker was attenuated upon pre-infusion with L-NAME.

**Conclusion:**

This study introduces a new approach to involve NO in physiology of aggregation of *Paramecia* following exposure to the misused sedative drug, morphine.

## INTRODUCTION

Influence of abused drugs in superior animals, the mammals, has been studied extensively (1, 2). Neuronal and neuroendocrine systems have also been involved in the response of higher animals to the misuse of drugs (3, 4).

Nitric oxide (NO), a molecule which participates in a variety of physiological functions such as neuronal transmission and cell-cell signaling in the nervous system (5), blood pressure and behavior regulation (6), has been implicated in seeking behavior of the opioids (7, 8). As an important regulator of mammalian physiology (6), the NO, can be produced by the interaction of the amino acid arginine and the enzyme nitric oxide synthase (NOS) (9). NO producing enzyme, the NOS, was initially described in isolated macrophages and endothelial cells (10). Thus far, NOSs have been described in invertebrates (11) and, more recently, in protists (12, 13). Production of NO in *P. caudatum* has been explained much more recently by Malvin *et al*. (2003) (14).

Free-living invertebrates contain the opioid receptor types as been reported previously (15–17). The opioid receptors in the simple animals are indicated as mediator in processing of dopamine and NO (18, 19). A µ-opiate receptor subtype in *Mytilus* is defined as the receptor with a high percent (95%) sequence identity to that of human neuronal mu-opiate receptor (15).

As much the literature as been reviewed no evidence provides that *P. caudatum* is amongst the organisms nominated as sensitive to the abuse substance, morphine. To discuss the presence of opioid receptor in *Paramecium* this team aimed to survey on the contact of single-celled animal model *Paramecium* to the misuse drug morphine. Also we researched on the role of NO in response of *Paramecium* to the morphine exposure. Furthermore, the activation of NADPH-diaphorase (NADPH-d), an established cytochemical marker of NOS in the living systems (20) was measured to explain the expression of NOS in the little organism. This study is the first that explains the physiology of protozoa in contact to narcotic misuse materials using the behavioral and biochemical assessment. Also we aimed to involve the NO in physiology of *P. caudatum*.

## MATERIALS AND METHODS

### Subject (Collection, Identification, and Cultivation)

**Fig. 1:**
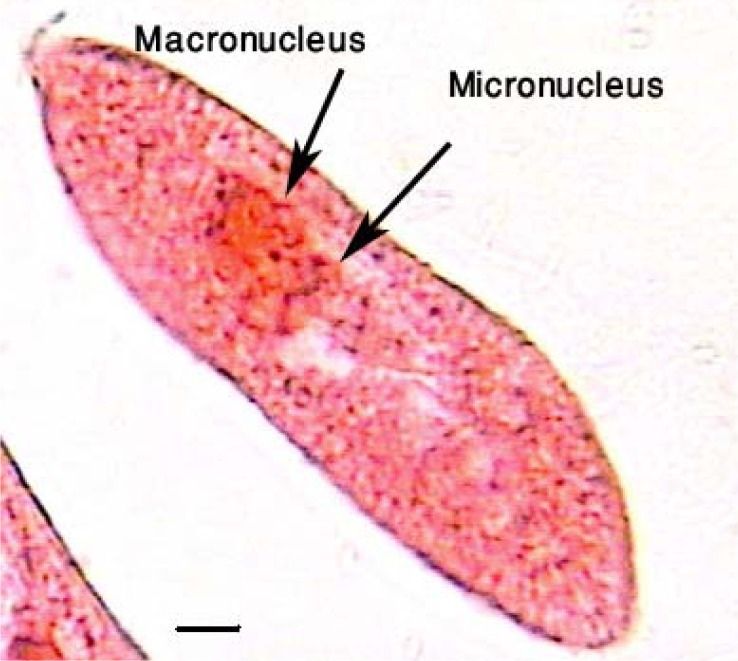
This figure shows the *P. caudatum* under the Feulgen's nuclear reaction. As the figure provides the nuclei (macronucleus & micronucleus) were highlighted as accordingly as previously been identified specifically (22).

**Fig. 2:**
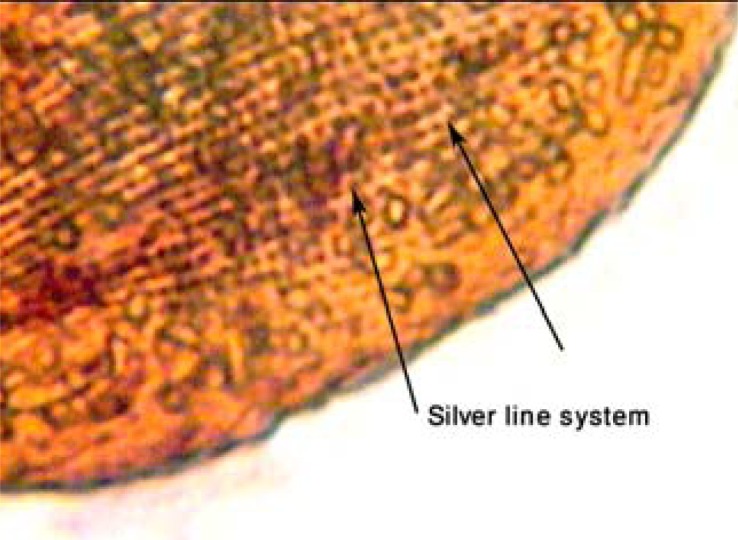
*P. caudatum* under the Klien's dry silver method. The silver line system was indicated as accordingly as been specifically identified previously (22).

Organisms were collected from the temporary fresh water bodies in the Tehran region, including small bogs and ponds using the collection map previously been reported (21). The collected samples were promptly examined under the microscopic magnification to find the desired organism. To determine specifically the microorganisms they were massively studied using the Feulgen's nuclear reaction and Klien's dry silver method in accord with the evidence been mentioned previously (22). Beside the evidence (Figs. 1 – 2) the pattern of the movement were evaluated in the free-swimming organisms. To slow the moving organisms a few cotton fibers were added to the sample. The organisms were furthermore examined biometrically. The identified *Paramecia* were cultivated in the natural polyculture medium (hay infusions: 10 g/l of tap water at 19 to 21°C). Hay infusions were boiled for about 5 min; the boiled hay was then allowed to settle and the supernatant was used as a culture medium (23).

The isolated organisms were also cultivated in a specific medium. This enriched medium was prepared by means of gradients and concentrations described in the manuscript (refer to section 2.2). Each sample (20 ml) of this medium was incubated at 29 to 31°C for about 48 hr.

It should be noted that the fresh-water protozoa of the Tehran region have already been classified and recorded by this laboratory (24).

### Growth of organisms in specific medium

*P. caudatum* was cultivated in fresh specific medium containing *Saccharomyces cerevisiae*, a species of budding yeast (2 g/l of sterile distilled water), which was enriched by salts (0.2 g/l CaCl_2_ and 0.2 g/l MgCl_2_). This medium was adjusted to a pH of 6.8 ± 0.2 and was maintained at 29 to 31°C.

### Drugs

Morphine sulphate (TEMAD, Co., Tehran, Iran), L-arginine (Sigma Chemical Co., USA) and N^G^-Nitro-L-arginine Methyl Ester (L-NAME; Research Biochemical Inc., USA) were prepared fresh in sterile distilled water (vehicle). The chemical gradients were purchased from Merck Chemicals (Germany).

### Microscopic counts of protozoa

The population of *P. caudatum* was counted daily using a Sedgwick-Rafter cell counting chamber (Graticules, Ltd., UK), a useful device for studying the growth of microorganisms. This chamber, holding a little more than 1 ml, can be covered with a thin coverslip, allowing microscopic examination with objectives up to 16X. The cells were counted by light microscopy using a 4X objective. A sample of the cell culture was placed in the chamber, which was then covered with a coverslip. The microorganisms were subsequently allowed to settle in the chamber. This step was performed as quickly as possible to ensure that the protozoa were randomly distributed and settled uniformly in the chamber (23, 25). The samples without adding of speed reducer (e.g. cotton fibers) were accurately diluted to provide a definite number of cells per view throughout the experiments. This step was repeatedly performed (at least 5 times) and the number of the organisms was counted in each view by quickly looking of the area. The mean of the counts was then calculated and reported. To minimize the visual errors an image of each viewing area was also taken. Cell populations were counted in 100-µm^2^ units of the images by an Olympus light photomicroscope at 4X magnification. Furthermore, the Image Tool program (UTHSCSA, version 2.03), the free image processing and analysis program for Microsoft Windows, was used for image analysis after spatial calibrations to provide quantification for an area of 100 µm^2^.

### Measurement of morphine potency *in Paramecium caudatum*

To measure the potency of morphine in *P. caudatum* the experiments were designed and performed as follows:

### Pretest phase

Initially, a sample (1 ml of cultivation medium) of the cells was placed in the chamber; permitting cell viability for about 30 min (23). The cells were placed in the midline of the apparatus and allowed free access to the entire apparatus for 5 sec. The number of cells in the chamber was counted at low power (4X objective) using the protocol described above. In addition, all events were recorded by photo-video microscopy (Olympus), and later the records were reviewed by a blind observer. The data were finally analyzed with the Image Tool program.

### Potency phase

**Fig. 3:**
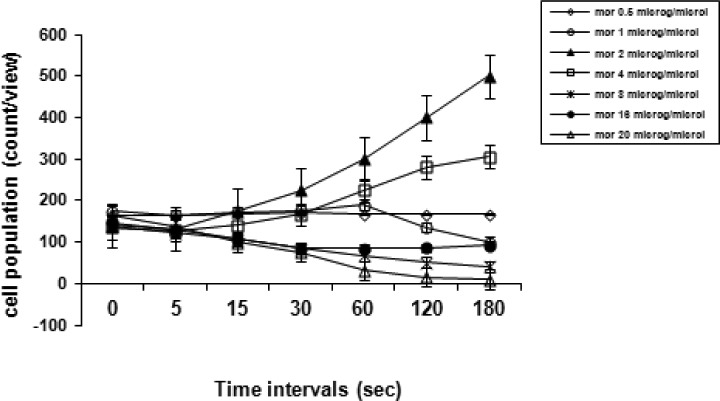
This fig. shows the effect of morphine at different concentrations (0.5 to 20 µg/µ1) upon infusion into the chamber. All procedure was as adjusted as detailed in the sections 2-5-1 and 2-5-2. The response to morphine was assessed by analysis of variance (ANOVA), and a definite testing point (60 sec) was chosen for subsequent experiments. Each time point provides the data as mean ± S.E.M.

This phase was started after the pretesting phase, which is also considered step one (the familiarization phase with a length up to 5 sec). This phase was promptly initiated with the infusion of morphine (0.5 to 60 µg/µ1) (Fig. 3); thus, the pre-test phase was switched to the step two (potency phase), which lasted from 5 to 180 sec by drug infusion (the morphine-pairing session). A corner of the apparatus was chosen for infusion of drugs. Drug infusions at the desired concentrations (in a total volume of 1 µ1) were performed by a glass 1- µ1 Hamilton syringe as accurately as possible over a definite time period. Control groups solely received distilled water (1 µ1).

### Testing phase

Cell aggregation was measured (counts/view) during the potency phase 60 sec after morphine infusion.

### NADPH cytochemistry

After testing, the experimental samples were fixed to provide adequate NADPH staining (26). The protocol, with little modification, was used to prepare specimens for examination by light microscopy. The protocol was also applied to follow routine protocols (clarification, dehydration) to perform NADPH cytochemistry. The slides were rinsed with buffer and then stained using the NADPH-diaphorase (NADPH-d) technique to visualize NOS activity. Briefly, the prepared slides were incubated with shaking in a 0.3% Triton-X 100 in phosphate buffer for 1 to 2 min. The staining was then performed by incubating the slides in a solution containing equal parts of nitro-blue tetrazolium (NBT, 0.2 mg/ml in buffer) and NADPH (1 mg/ml in buffer) for about 30 min at 37°C. Upon reduction by NADPH-d, NBT yields a blue formazan that is visible by light microscopy (26, 27). No staining was observed in negative control samples incubated without NADPH.

### Statistical analysis

Data are presented as mean± SEM. Groups were compared using one-way analysis of variance (ANOVA). Differences between groups were measured by means of Tukey-Kramer post-hoc test. A p-value of < 0.05 was the threshold for statistical significance. The NADPH-diaphorase (NADPH-d) reaction in a 100-µm^2^ area was examined in the samples (7 samples) of each experiment by a light microscope (Olympus). Intensity analysis was assessed in 100-µm^2^ units using the photorecords at 4X magnification with the Image Tool program (UTHSCSA, version 2.03), the free image processing and analysis program for Microsoft Windows for quantification analysis for an area 100 µm^2^.

## RESULTS

### Cell growth in specific medium (48 h)

**Fig. 4:**
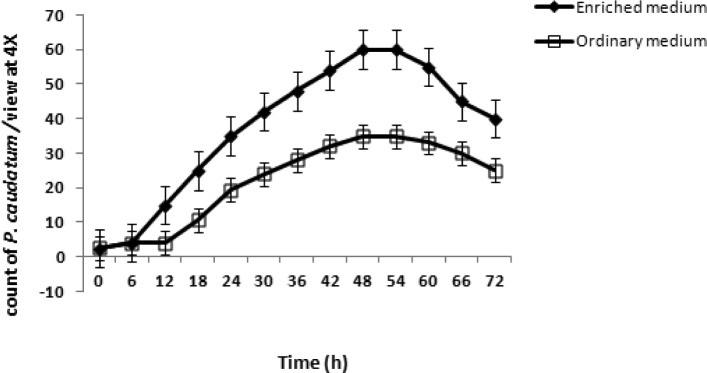
This fig. shows the results of the cultivation of *P. caudatum* in a specific enriched medium specified in the Materials and Methods. Population of *Paramecia* in the medium showed an increase during 48 hr ranging from 266 ± 9 to 5999 ± 51 (numbers of cells/ml). Through the ascending phase of the growth phase a significant increase in the cell population occurred from 6 hr up to 48 hr.

*P. caudatum* was cultivated in a specific enriched medium detailed in the Materials and Methods. The number of *Paramecia* in the medium showed an increase during 48 h ranging from 266 ± 9 to 5999 ± 51 (number of cells/ml). Throughout the ascending phase of the growth phase, a significant increase in the number of *Paramecia* occurred from 6 hr up to 48 hr (Fig. 4).

### Induction of morphine potency in *P. caudatum*

**Fig. 5:**
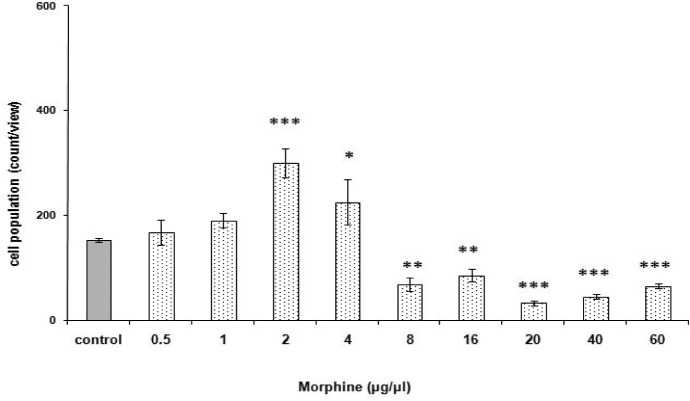
Dose response curve for morphine in *P. caudatum*. Different doses of morphine (0.5 to 60 µg/µ1) or distilled water (1 µ1) were given in accord with the program detailed in the Materials and Methods. Score is defined as the cell counts/view at the time point 60-sec and is expressed as mean score ± S.E.M. *p < 0.05, *p < 0.01, ***p < 0.001 differences to the control group based on analysis by Tukey-Kramer.

Fig. 5. shows a morphine potency curve in *P. caudatum* with a protocol developed in our laboratory. The opioid induced a dose-dependent significant response [F (9, 45) = 49.700, *p < 0.0001*] over time (sec). The maximum morphine-induced increase in *Paramecia* numbers occurred at 2 µg/µ1 of the drug. Thus, this dose was used for the subsequent behavioral tests.

### Effect of L-arginine (NO precursor) on morphine aggregation in *P. caudatum*

**Fig. 6:**
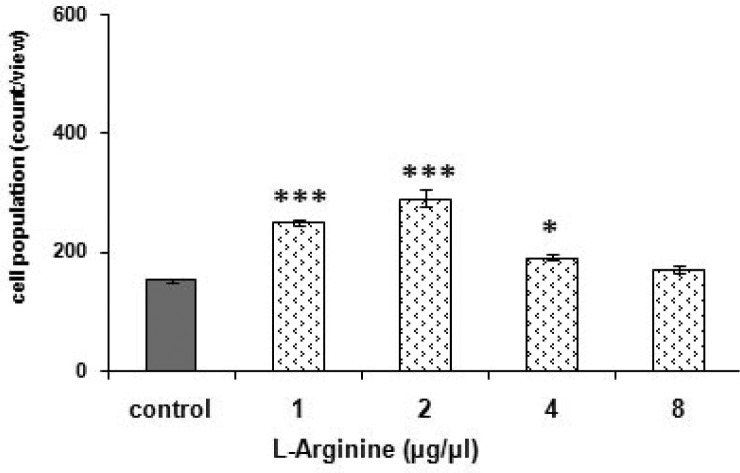
Effects of L-arginine on cell aggregation. Cells received 1 µ1 distilled water (grouped as the control group) or solely L-arginine (1 to 8 µg/µ1). Score is defined as been noted in Fig. 3. * *p < 0.05*, ***p < 0.001 differences to the control group based on analysis by Tukey-Kramer.

**Fig. 7:**
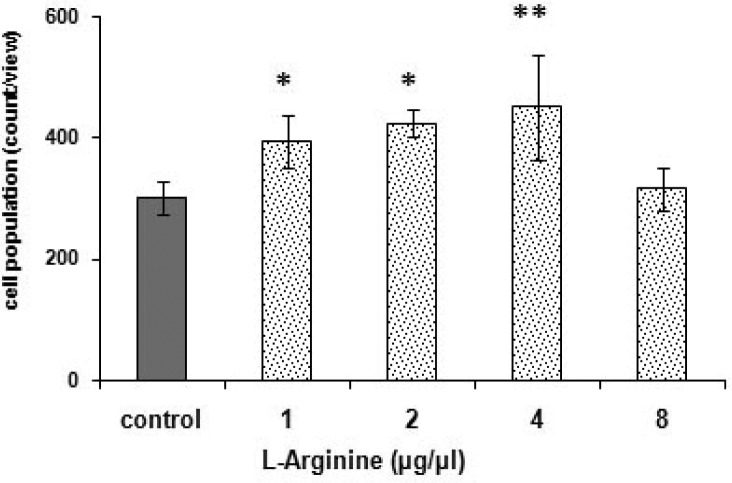
Effects of L-arginine with morphine on cell aggregation. Cells received distilled water (1 µ1) (control) or L-arginine (1 to 8 µg/µ1) in the presence of morphine (2 µg/µ1). Score is defined as been described in Fig. 3. * *p < 0.05*, **p < 0.01 differences to the control group based on analysis by Tukey-Kramer.

Fig. 7. shows the significant [F (4, 20) = 4.326, *p < 0.05*] effect of L-arginine, the NO precursor, on morphine accumulation potency in *P. caudatum*. The NO-generating agent potentiated the morphine response in a dose-dependent manner. Based on the results, a dose of 4 µg/µ1 of L-arginine was used for subsequent behavioral testing. Notably, L-arginine itself showed also a significant response [F (4, 20) = 156.629, *p < 0.0001*]. The most effective single dose of the agent was 2 µg/µ1 according to the post-hoc Tukey-Kramer test (Fig. 6.).

### Effect of pre-infusion of L-NAME on potentiating of aggregation of *P. caudatum* due to morphine

**Fig. 8:**
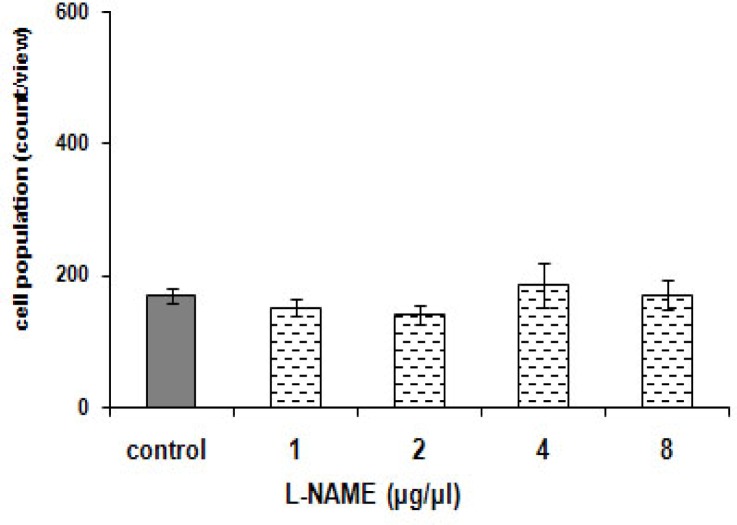
Effect of L-NAME on *Paramecia*. Cells were treated with distilled water (1 µ1) (control) or single L-NAME (1 to 8 µg/µ). Score is calculated as been explained in Fig. 3. No significant effect was recorded.

**Fig. 9:**
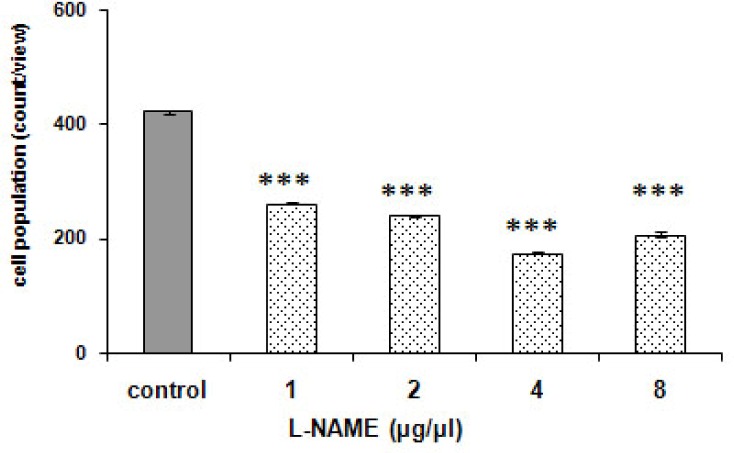
Effect of L-NAME on response to L-arginine together with morphine in *Paramecia*. Cells were treated with distilled water (1 µ1) (control) or L-NAME (1 to 8 µg/µ1); the cells were treated distilled water or L-NAME (1 to 8 µg/µ1) prior to L-arginine (4 µg/µ1) in the presence of morphine (2 µg/µ1). Score is obtained as been defined in Fig. 3. ***p < 0.001 significant difference to the control distilled water before L-arginine plus morphine based on analysis by Tukey-Kramer.

Fig. 9. reveals the effect of L-NAME, the NOS inhibitor, on L-arginine plus morphine- aggregation of *P. caudatum*. The effect of L-NAME was statistically significant [F (4, 20) = 2118.420, *p < 0.0001*). The NOS inhibitor blocked the observed effect in a manner independent on the dose. Though, the agent caused no significant response by itself (*p > 0.05*) (Fig. 8).

### Nitric oxide production (activation of NOS)

**Fig. 10:**
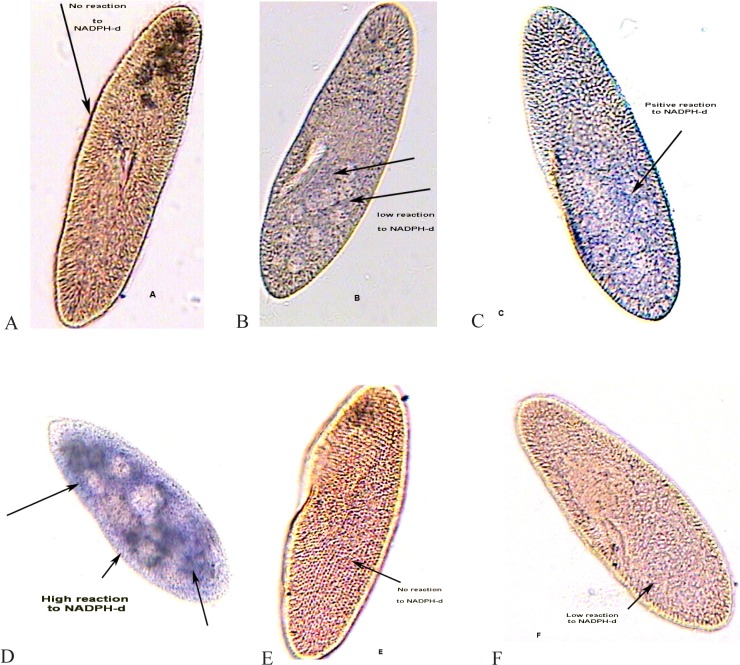
Positive NADPH-diaphorase (NADPH-d) cytochemistry in samples treated with L-arginine prior to morphine (10D). The figure also shows the reaction to NADPH-d in samples treated with L-NAME prior to L-arginine plus morphine (10F). 10A: negative control (distilled water); 10B: positive control (morphine only); 10C; another control (L-arginine only); 10E: another control (L-NAME only). Arrowhead shows the positive area for NADPH-d.

**Fig. 11:**
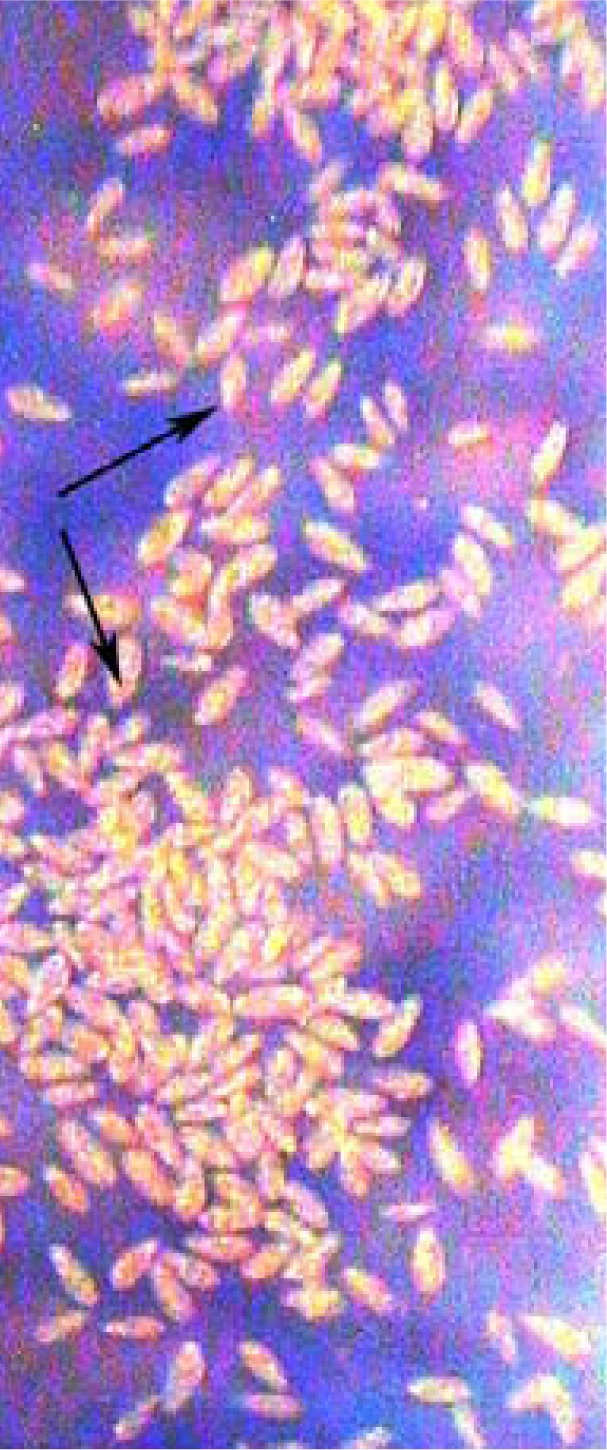
This figure shows the accumulation of *P.caudatum* under a fixed view (4X) of light microscopy. The figure shows the sample after drug infusion using the schedule detailed in the Materials & Methods.

Fig. 10. shows the activated NO synthase (NOS) (*p < 0.01*) under infusion of L-arginine the NO precursor. The NO agent caused an activation of the NOS, a process leading to NO production and confirming that the NO is participated in the cell aggregation (Fig. 11).

### NOS inhibition

As Fig. 10. shows, the pre-infusion of L-NAME before of infusion of L-arginine, caused an inhibition of NOS at a statistically significant level (*p < 0.01*). The antagonist reversed the effect of the NO precursor L-arginine confirming the NO involvement in accumulation of *Paramecia* (Fig. 11).

## DISCUSSION

As the main results of this study show: (1) morphine exhibited potency for the aggregation of *P. caudatum*, (2) after infusion of L-arginine and most likely due to nitric oxide (NO) production the microorganisms accumulated more potently, (3) L-NAME reversed the potentiating effect of L-arginine on morphine influence, (4) L-arginine plus morphine significantly increased NADPH-diaphorase (NADPH-d) expression while the expression of the marker grew less after the usage of L-NAME.

Previous data have shown that *P. caudatum* produces NO, and that L-NAME inhibits the NO production (14). Malvin *et al*. (2003) (14) also suggested that *P. caudatum* produces NO from L-arginine by a calcium-sensitive NO synthase (NOS). A role for L-arginine in NO production has been confirmed in a later study by examining the ability of *P. caudatum* to produce [3H] L-citrulline from [3H] L-arginine via a mechanism inhibited by L-NAME.

The present results may demonstrate for the first time that the molecule NO is participated in attracting of *P. caudatum* to narcotic drug. This study examined the possible role of the NO system in the unicellular animal *P. caudatum* in response to the abuse drug morphine. We showed that the aggregation of the cells in contact to morphine was enhanced in the animals pre-infused with the NO precursor, L-arginine. The NO system, therefore, may be involved in morphine-induced aggregation in the single-celled animal *P. caudatum*.

Thus, this organism may be usable as a new model for measuring of morphine potency in living organisms. Indeed, because that this unicellular model is low-cost and easy to manipulate, it is acquiescent that be used to interrogate the cellular mechanisms governing on opioid dependence.

The unicellular organisms, *Paramecia*, as been previously demonstrated express several NO-sensitive targets, including guanylyl cyclase, potassium channels and voltage gated calcium channels (28–32). Other studies using the ciliate protozoan *Stentor have* demonstrated that these types of microorganisms express a G protein-mediated response to morphine when stimulated mechanically (33).

The NO which has been classified as an important signaling molecule that plays role in various physiological processes in invertebrates (34) is also implicated in pathogenesis of several diseases in vertebrates. Previous results have also implicated the molecule NO in invertebrates as a main regulator of release of transmitter acetylcholine (35).

To quantitatively identify the mechanisms governing on morphine-induced aggregation of *P. caudatum*, the results of NADPH-d were analyzed that properly support this detail. The fact is that the NO signals the aggregation response to morphine in the single-celled organism, *P. caudatum*. The NO which has been introduced as a molecule of great pharmacological interest and physiological importance (9) participates in a variety of the mammalian physiological functions (6). The molecule has been implicated in morphine-induced rewarding (36, 37) and morphine-induced pain reduction (38). This work moreover contributes the NO in morphine-induced aggregation in the single celled animal *P. caudatum*, the response which is likely mediated by the opioid receptor- and the NO-dependent cell signaling pathways.

In conclusion, the contribution of molecule NO in morphine-induced aggregation of *P. caudatum* was shown by using a novel behavioral assay. This work may properly involve the NO in the functioning of the microorganism to misuse drug exposure.
